# Antibiotic Resistance, Biofilm Formation, and Molecular Epidemiology of Foodborne *Staphylococcus aureus* Isolated in Northwest Hubei Province, China

**DOI:** 10.1002/fsn3.70791

**Published:** 2025-08-16

**Authors:** Yitong Tang, Yanyan Li, Na Xiao, Lu Wang, Shichao Li, JiuMing Zou, Yanmin Yu, Jinhui Shao, Ming Jiang

**Affiliations:** ^1^ Medicine College Hubei University of Arts and Science Xiangyang China; ^2^ Medicine College Pingdingshan University Pingdingshan China; ^3^ Xiangyang Central Hospital The Affiliated Hospital of Hubei University of Arts and Science Xiangyang China

**Keywords:** antimicrobial susceptibility, biofilm, foodborne, multilocus sequence typing, *Staphylococcus aureus*, virulence genes

## Abstract

*Staphylococcus aureus* is a common pathogen responsible for foodborne infections worldwide. This study investigated the antibiotic resistance profiles, biofilm formation capacity, and molecular epidemiological characteristics of foodborne *S. aureus* isolates from northwest Hubei Province, China, as well as the correlation among these factors. Among the 303 food samples collected from Xiangyang, Suizhou, and Shiyan cities, 41 yielded non‐duplicate *S. aureus* strains. Of the 41 *S. aureus* isolates, 8 (19.51%) were identified as MRSA, while 33 (80.49%) were methicillin‐susceptible *S. aureus* (MSSA). High resistance was observed to penicillin (78.05%), tetracycline (43.90%), and erythromycin (31.71%), with MRSA strains demonstrating significantly stronger resistance profiles than MSSA strains. Among MRSA isolates, 50% (4/8) demonstrated strong biofilm‐forming capacity, compared to only 9.09% (3/33) of MSSA isolates. Strong biofilm formation was observed more frequently in isolates from frozen meat (66.67%, 4/6) than in those from vegetarian salads (0%, 0/9) or fresh meat (12.50%, 2/16). The prevalence of Panton‐Valentine leukocidin (PVL) gene was higher in MRSA strains, whereas enterotoxin genes were more commonly found in MSSA strains, though differences between groups were not statistically significant. The primary epidemic clones identified were CC88‐ST88‐t1376/t437, CC7‐ST7‐t091/t3884, and CC5‐ST6/ST462‐t701/t165, constituting 63.41% (26/41) of isolates. The CC59‐ST59/338 strain exhibited a pronounced capacity for strong biofilm formation. SCCmecII‐CC15‐ST15‐t085 and SCCmecIII‐CC7‐ST7‐t3884 strains exhibited the highest antibiotic resistance, with resistance to 9 and 7 antibiotics, whereas CC88‐ST88‐t1376, CC7‐ST7‐t091, and CC5‐ST6‐t701 showed resistance to fewer than three antibiotics. The findings enhance the understanding of the drug resistance profiles and molecular epidemiology of foodborne *S. aureus* , providing a foundation for more effective control measures.

## Graphical abstract

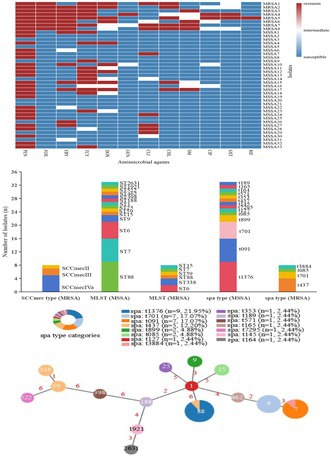

## 1 Introduction

*Staphylococcus aureus* is a prevalent foodborne pathogen capable of contaminating food and producing enterotoxins that lead to food poisoning symptoms, such as nausea, vomiting, and, in severe cases, toxic shock syndrome, posing a significant threat to food safety worldwide (Macori et al. 2019; Ou et al. 2020; Schneewind and Missiakas 2019; Jenul and Horswill 2019). In 2018, *S. aureus* was one of the top 10 pathogens responsible for the highest number of hospitalizations in foodborne and waterborne outbreaks across 36 European countries (EFSA and ECDC 2019). According to the Centers for Disease Control and Prevention in the United States, *S. aureus* ranks sixth among bacterial causes of foodborne outbreaks (Centers for Disease Control and Prevention 2019). Data from the Chinese Foodborne Disease Outbreak Monitoring System reported that *S. aureus* was the third most common cause of bacterial foodborne outbreaks in 2020, following *Salmonella* and *Vibrio parahaemolyticus* (Li et al. 2021).

Biofilm formation is a critical virulence factor for *S. aureus* , playing an essential role in infection persistence and resilience (Tam and Torres 2019). Biofilms provide a protective environment that shields the bacteria from host immune responses and antimicrobial agents, thus enhancing *S. aureus* resistance, pathogenicity, and immune evasion capabilities. The formation of biofilms by *S. aureus* in food processing facilities and equipment creates a persistent contamination risk, as these biofilms are difficult to remove and pose potential hazards to food safety (Hernández‐Cuellar et al. 2023). Monitoring biofilm formation by foodborne *S. aureus* at various stages of food production and storage, as well as developing strategies to eliminate these biofilms, are critical for preventing *S. aureus* contamination and foodborne infection.

Antibiotic resistance in *S. aureus* , especially methicillin‐resistant *S. aureus* (MRSA), has escalated due to extensive antibiotic use. MRSA strains, harboring multiple resistance genes, are often resistant to various antibiotics, complicating clinical treatment, increasing healthcare costs, and even raising mortality rates associated with infection (Thampi et al. 2015). In 2017, the World Health Organization identified MRSA as one of 12 bacteria posing the highest threats to human health due to antibiotic resistance (Willyard 2017). MRSA is now prevalent globally (Jin et al. 2021; Bavaro et al. 2024). Furthermore, differences in antibiotic use across regions and over time have led to dynamic shifts in the molecular epidemiology and resistance profiles of *S. aureus* , adding complexity to efforts in epidemic prevention and control (Madera et al. 2023; Chen et al. 2021). Therefore, continual monitoring of the prevalence, virulence, and antibiotic resistance profiles of foodborne *S. aureus* strains is crucial, especially for developing timely intervention strategies to mitigate foodborne infections (Hou et al. 2023).

This study investigated the antibiotic resistance, biofilm formation capabilities, virulence gene profiles, and molecular epidemiological characteristics of foodborne *S. aureus* isolates from northwest Hubei Province, China. Additionally, we evaluated the correlation among these factors to provide a scientific foundation for more effective control of foodborne *S. aureus* infections and to support future epidemic prevention efforts.

## 2 Materials and Methods

### 2.1 Source and Identification of Bacterial Strains

Forty‐one *S. aureus* strains were isolated from 303 food samples (13.53%, 41/303) collected between April and December 2018 from large supermarkets, farmers' markets, and individual stalls in northwest Hubei Province (Xiangyang, Suizhou, and Shiyan cities). These isolates included: 16 strains (24.62%, 16/65) from fresh meat products, 6 strains (22.22%, 6/27) from frozen meat products, 4 strains (6.45%, 4/62) from cooked meat products, 9 strains (14.75%, 9/61) from vegetarian salads, 2 strains (5.56%, 2/36) from rice and noodle products, and 4 strains (9.09%, 4/44) from bean products. No strains were isolated from 8 samples of fruits and cold drinks. To avoid duplicate isolates, only one sample was collected from each sampling location.

Food samples were processed following the GB/T 4789.10‐2016 standard (National Standard of the People's Republic of China 2016). Samples were pre‐enriched in trypticase soy broth (TSB) (Guangdong Huankai Microbial Technology Co. Ltd., Guangzhou, China) and then cultured on Baird‐Parker medium for preliminary screening and isolation of *S. aureus* . Biochemical identification was performed through colony morphology, Gram staining, coagulase, and catalase testing. Bacterial DNA was extracted using the Rapid Bacterial Genomic DNA Isolation Kit (Sangon Biotech Co. Ltd., Shanghai, China), following the manufacturer's instructions. *S. aureus* isolates were confirmed by PCR amplification of the species‐specific *femA* gene (Haghi et al. 2021; da Silva et al. 2005). Isolates were designated as MRSA if the *mecA* gene was detected through PCR (Oliveira and de Lencastre 2002). ATCC25923, ATCC29213, and ATCC43300 were used as quality control strains throughout the study (Yang et al. 2022).

### 2.2 Determination of Antimicrobial Susceptibility

Antimicrobial susceptibility was determined using the disk diffusion method according to the guidelines of the Clinical and Laboratory Standards Institute Guidelines (CLSI 2023). Overnight cultures of each isolate were adjusted to a 0.5 McFarland standard and inoculated onto Mueller‐Hinton agar (HuanKai Microbial, China). Antimicrobial disks (Hangzhou Microbial Reagent Co. Ltd., HangZhou, China) included: penicillin (PEN) (10 UI), cefoxitin (FOX) (30 μg), erythromycin (ERY) (15 μg), gentamicin (GEN) (10 μg), tetracycline (TCY) (30 μg), doxycycline (DOX) (30 μg), ciprofloxacin (CIP) (5 μg), clindamycin (CLI) (2 μg), trimethoprim/sulfamethoxazole (SXT) (1.25/23.75 μg), streptomycin (SM) (10 μg), rifampicin (RIF) (5 μg), and chloramphenicol (CHL) (30 μg). After incubation at 37°C for 24 h, inhibition zone diameters were measured to determine susceptibility.

### 2.3 Biofilm Formation Assay

Biofilm formation capacity was assessed using the microplate assay (Hamad 2023; Tang et al. 2024). Each isolate was cultured overnight in TSB with 0.5% glucose, then diluted 1:40 in fresh TSB‐0.5% glucose (Sigma‐Aldrich Inc., St. Louis, Missouri, USA). Aliquots (200 μL) of the bacterial suspension were added to each well of a microtiter plate, while negative control wells received 200 μL of TSB‐0.5% glucose. The plates were incubated at 37°C for 48 h, followed by fixation with 200 μL methanol for 20 min. After drying at 37°C, wells were stained with 0.1% crystal violet for 10 min. Excess dye was removed, and 200 μL of 95% ethanol was added to dissolve the crystal violet bound to adherent cells. Absorbance (OD) was measured at 540 nm using an ELx800 ELISA Microplate reader (BioTek Instruments Inc., Vermont, USA).

The experiment was performed in triplicate, and the optical density cutoff (ODc) was calculated as the mean OD of the negative control plus three standard deviations (SD). Biofilm formation by various strains (ODs) was categorized as follows: strong biofilm formation if ODs > 4ODc; moderate if 2 ODc ≤ ODs ≤ 4 ODc; weak if ODc ≤ ODs ≤ 2 ODc; and no biofilm formation if ODs ≤ ODc.

### 2.4 Detection of Antibiotic Resistance and Virulence Factor Genes

The presence of antimicrobial resistance gene (*blaZ*), biofilm‐associated genes (*icaAD*, *icaBC*, *sasC*, *sasG*, *fnbA*, and *pls*), the Panton‐Valentine leukocidin (PVL) gene, and enterotoxin genes (*sea* to *see*) was assessed in all isolates using PCR, as described in previous studies (Ghabbour et al. 2022; Tang et al. 2013; Lina et al. 1999; Mehrotra et al. 2000). The primers utilized are detailed in Table 1. Reactions were carried out in a volume of 20 μL with a 2 × PCR Mastermix (Sangon Biotech, China), 0.5 μM of each primer, and 1 μL of purified sample DNA template. The mixtures were submitted to a program performed on a TaKaRa TP600 thermocycler (Takara Bio Inc., Japan). The initial denaturation step took place at 95°C for 5 min. A total of 35 amplification cycles were performed, each of which took 40 s at 95°C, 50 s at varying temperatures for different genes (Table 1), and 50 s at 72°C, followed by an additional extension step of 10 min at 72°C.

**TABLE 1:** PCR primer sequences of target genes.

Gene	Primers sequences (5′‐3′)	Products sizes (base pairs)	Tm
*blaZ*	F:TACAACTGTAATATCGGAGGG R:CATTACACTCTTGGCGGTTTC	833	50°C
*icaAD*	F: TGGCTACTGGGATACTGATA R: TGGAAATGCGACAAGAACTA	520	55°C
*icaBC*	F: GCCTATCCTTATGGCTTGA R: TGGAATCCGTCCCATCTC	182	56°C
*sasC*	F: AGAATGAAGTCCGATAGAGT R: AATCATACAGATGGCAATAC	936	53°C
*sasG*	F: TATCAACACTTCCGTAACCTTC R: CGTCAGTCACTCATAACGCAGA	159	59°C
*fnbpA*	F: TCCGCCGAACAACATACC R: TCAAGCACAAGGACCAAT	952	54°C
*pls*	F: ACACCAGCAGTTGAAGAC R: TATTGAATGCAGTTAGCG	442	55°C
*luk‐PVL*	F:ATCATTAGGTAAAATGTCTGGACATGATCCA R:GCATCAASTGTATTGGATAGCAAAAGC	433	55°C
*sea*	F:GGTTATCAATGTGCGGGTGG R: CGGCACTTTTTTCTCTTCGG	102	53°C
*seb*	F:GTATGGTGGTGTAACTGAGC R: CCAAATAGTGACGAGTTAGG	164	53°C
*sec*	F:AGATGAAGTAGTTGATGTGTATGG R: CACACTTTTAGAATCAACCG	451	54°C
*sed*	F:CCAATAATAGGAGAAAATAAAAG R: ATTGGTATTTTTTTTCGTTC	278	54°C
*see*	F:AGGTTTTTTCACAGGTCATCC R: CTTTTTTTTCTTCGGTCAATC	209	53°C

### 2.5 Staphylococcus Enterotoxin Detection

Staphylococcal enterotoxins (sea‐see) were detected using a commercial ELISA kit (Tianjin Yueteng Biotechnology Co. Ltd., China). *S. aureus* isolates were pre‐cultured in Tryptic Soy Broth (TSB) at 37°C for 48 h. Culture supernatants were collected by centrifugation (4000 × g, 10 min, 4°C) and analyzed following the manufacturer's protocol. Absorbance was measured at 450 nm using an ELx800 microplate reader.

### 2.6 SCCmec Typing

The SCCmec typing of MRSA strains was performed using multiplex PCR (Zhang et al. 2005, 2012). Specific primers targeting each SCCmec type and subtype were used for PCR amplification. Amplified products were separated by 1.2% agarose gel electrophoresis, and SCCmec types I–V were determined based on the distinct band patterns corresponding to each SCCmec type.

### 2.7 Multilocus Sequence Typing

Primer design and PCR amplification for the seven *S. aureus* housekeeping genes (*arcC*, *aroE*, *glpF*, *gmk*, *pta*, *tpi*, and *yqiL*) followed the protocols of the MLST database (https://pubmlst.org/). Each PCR product was sequenced, and the sequences were submitted to the MLST database for allelic identification, allowing determination of the sequence type (ST) for each strain. Strains sharing six or more identical allelic loci were grouped into clonal complexes (CC).

### 2.8 Spa Typing

Amplification of the *spa* gene was conducted using primers spa‐F (5′‐AGACGATCCTTCGGTGAGC‐3′) and spa‐R (5′‐GCTTTTGCAATGTCATTTACTG‐3′), as specified in the Ridom SpaServer database (http://spa.ridom.de/). Amplified products were sequenced and aligned to the repeat sequence database in Ridom SpaServer to determine the *spa* type of each isolate.

### 2.9 Statistical Analysis

Data were processed using SPSS Statistics 19.0.1. Categorical data are expressed as the number and percentage of bacterial strains, with statistical significance evaluated through Pearson's chi‐square test, continuity correction, or Fisher's exact test as appropriate. Significance was defined as *p* < 0.05. Minimum spanning tree analysis was performed using the goeBURST algorithm in the online phyloviz platform (http://online.phyloviz.net/index).

## 3 Results

### 3.1 Antibiotic Resistance

All isolates were tested for antimicrobial resistance, and the overall resistance rates for each antibiotic were as follows: penicillin (78.05%), tetracycline (43.90%), erythromycin (31.71%), doxycycline (21.95%), cefoxitin and clindamycin (19.51%), chloramphenicol (17.07%), streptomycin (12.20%), ciprofloxacin and trimethoprim/sulfamethoxazole (4.88%), and gentamicin and rifampicin (2.44%). Intermediate resistance was observed in 17.07% of isolates for doxycycline, 12.20% for ciprofloxacin, 9.76% for streptomycin, 7.32% for erythromycin, clindamycin, and trimethoprim/sulfamethoxazole, 4.88% for tetracycline and gentamicin, and 2.44% for chloramphenicol.

**FIGURE 1:**
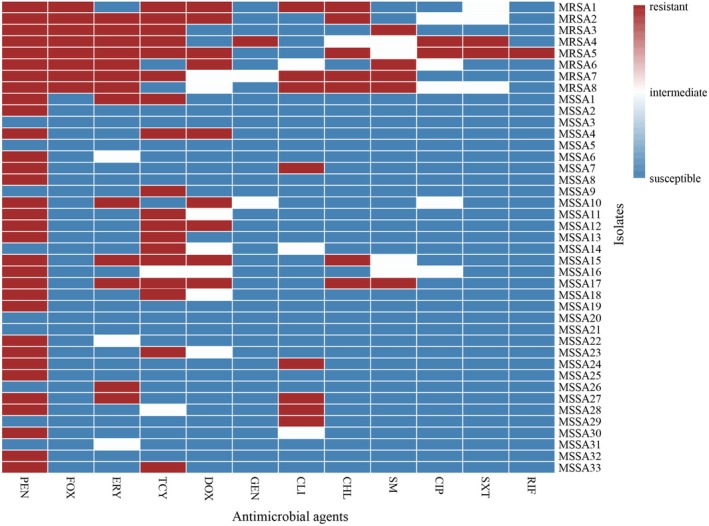
Heatmap of antimicrobial resistance patterns in MRSA and MSSA isolates. The heatmap was generated using R version 4.0.3. *X*‐axis: Tested antimicrobial agents; *Y*‐axis: Bacterial isolates grouped by strain type (MRSA/MSSA). Resistance color‐coding: Susceptible (blue), intermediate (white), resistant (red).

Among the 41 *S. aureus* isolates, 8 (19.51%) were identified as MRSA. All MRSA strains exhibited resistance to at least three categories of antimicrobials, classifying them as multidrug‐resistant (Liang et al. 2023). Additionally, five MSSA strains (15.15%, 5/33) were identified as multidrug‐resistant. Figure 1 compares the resistance patterns of MRSA and MSSA strains, showing that MRSA strains had higher resistance rates across all antibiotics compared to MSSA strains. MRSA strains demonstrated resistance rates exceeding 75% for penicillin, erythromycin, and tetracycline, and over 50% for chloramphenicol, doxycycline, and streptomycin. Conversely, MSSA strains showed moderate resistance to tetracycline, erythromycin, doxycycline, and clindamycin, with resistance rates ranging between 15% and 37%. MRSA strains exhibited significantly higher resistance rates to cefoxitin, erythromycin, chloramphenicol, streptomycin, ciprofloxacin, and trimethoprim/sulfamethoxazole compared to MSSA strains (*p* < 0.05). These findings indicate that foodborne MRSA strains in this region display robust resistance, while foodborne MSSA strains show moderate resistance to select antibiotics but remain largely sensitive to other antimicrobial agents.

### 3.2 Food Source and Distribution of Bacterial Strains

**FIGURE 2:**
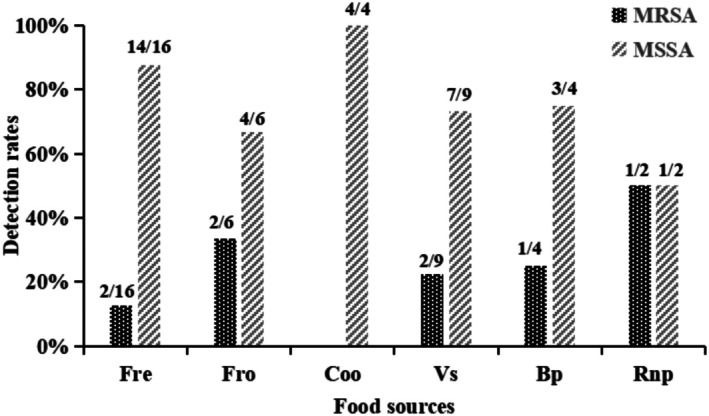
Prevalence of MRSA and MSSA isolates across food categories in Northwest Hubei Province, China. Food sources: Fresh meat (Fre, *n* = 16), frozen meat (Fro, *n* = 6), cooked meat products (Coo, *n* = 4), vegetarian salads (VS, *n* = 9), bean products (Bp, *n* = 4), rice and noodle products (Rnp, *n* = 2). Bars represent detection rates (%) of methicillin‐resistant (MRSA) and methicillin‐susceptible (MSSA) *Staphylococcus aureus* strains (total isolates: *N* = 41).

Among the 41 isolates, 16 strains (39.02%) were from fresh meat, 6 (14.63%) from frozen meat, 4 (9.76%) from cooked meat, 9 (21.95%) from vegetarian salads, 4 (9.76%) from bean products, and 2 (4.88%) from rice and noodle products. The distribution of MRSA and MSSA strains across these food sources is presented in Figure 2. The highest raw proportions of MRSA were observed in rice/noodle products (50.00%, 1/2) and frozen meat (33.33%, 2/6); though small sample sizes preclude robust statistical comparison.

### 3.3 Biofilm Formation Analysis

As shown in Table 2, all isolates demonstrated the ability to form biofilms, with 17.07% (*n* = 7) categorized as strong biofilm producers, 46.34% (*n* = 19) as moderate, and 36.59% (*n* = 15) as weak biofilm formers. Notably, MRSA strains showed a strong biofilm formation rate of 50%, which was significantly higher than that of MSSA strains at 9.09% (*p* < 0.05).

**TABLE 2:** Biofilm production by *S. aureus* isolates using the microtiter plate method.

Biofilm formation status	MRSA (*n* = 8)	Percent (%)	MSSA (*n* = 33)	Percent (%)	Total (*n* = 41)	Percent (%)	*p*
Strong	4	50.00	3	9.09	7	17.07	0.025^a^
Moderate	3	37.50	16	48.48	19	46.34	0.870^a^
Weak	1	12.50	14	42.42	15	36.59	0.243^a^

*Note:* ^a^Indicate biofilm formation of MRSA and MSSA by continuity correction (two‐sided).

The biofilm formation abilities of isolates from various food sources are presented in Table 3. Isolates from frozen meat demonstrated a significantly higher rate of strong biofilm formation (66.67%, 4/6) compared to isolates from fresh meat (12.50%, 2/16) and vegetarian salads (0%, 0/9) (*p* < 0.05). In contrast, isolates from cooked meat exhibited a significantly higher rate of weak biofilm formation (100%, 4/4) compared to isolates from frozen meat (0%, 0/6) and vegetarian salads (22.22%, 2/9) (*p* < 0.05).

**TABLE 3:** Biofilm formation ability of *S. aureus* isolates from different food sources.

Biofilm formation status	Fresh meat (*N*, %)	Frozen meat (*N*, %)	Cooked meat (*N*, %)	Vegetarian salads (*N*, %)	Bean products (*N*, %)	Rice and noodle products (*N*, %)	*p*
Strong	2 (12.50)	4 (66.67)	0	0	1 (25.00)	0	0.026^a^
Moderate	8 (50.00)	2 (33.33)	0	7 (77.78)	1 (25.00)	1 (50.00)	0.128^a^
Weak	6 (37.50)	0	4 (100)	2 (22.22)	2 (50.00)	1 (50.00)	0.023^a^

*Note:* ^a^Indicates biofilm formation of isolates from different food sources as determined by Fisher's exact test.

### 3.4 Distribution of Resistance and Virulence‐Associated Genes

**FIGURE 3:**
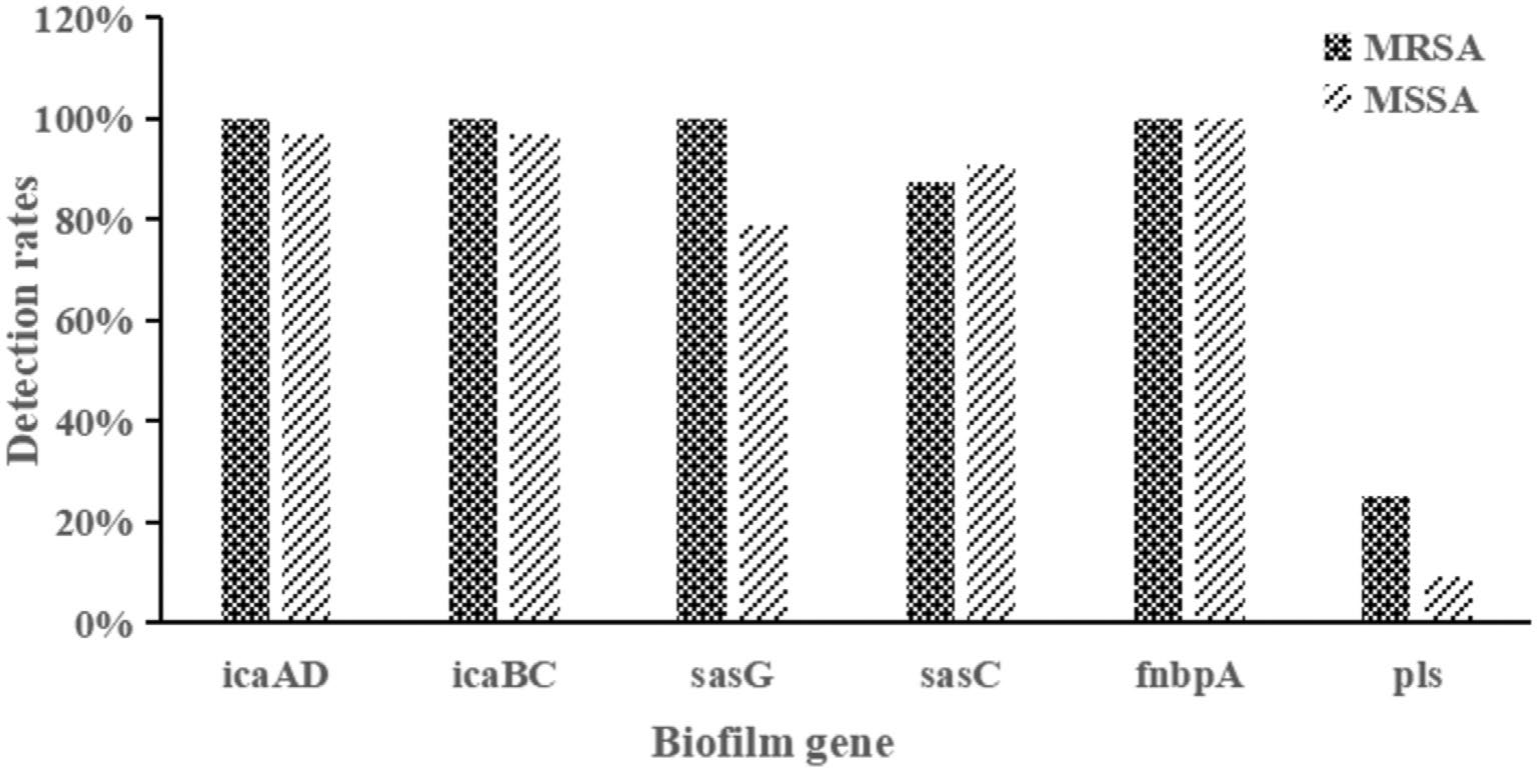
Comparative prevalence of biofilm‐associated genes in food‐derived MRSA versus MSSA isolates from Northwest Hubei, China. Bar chart showing PCR‐based detection rates (%) of adhesion genes: *icaAD*, *icaBC*, *SasG*, *SasC*, *fnbpA*, and *pls*. Columns represent methicillin‐resistant (MRSA, *n* = 8) and methicillin‐susceptible (MSSA, *n* = 33) *Staphylococcus aureus* strains.

As shown in Figure 3, the detection rates of biofilm‐related genes (*icaAD*, *icaBC*, *sasG*, *sasC*, *fnbA*, and *pls*) among MRSA strains were as follows: *icaAD* (100%, 8/8), *icaBC* (100%, 8/8), *sasG* (100%, 8/8), *sasC* (87.50%, 7/8), *fnbA* (100%, 8/8), and *pls* (25.00%, 2/8). In comparison, detection rates among MSSA strains were *icaAD* (96.97%, 32/33), *icaBC* (96.97%, 32/33), *sasG* (78.79%, 26/33), *sasC* (90.91%, 30/33), *fnbA* (100%, 33/33), and *pls* (9.09%, 3/33). Although MRSA strains exhibited higher detection rates for most biofilm‐related genes compared to MSSA strains (except for *sasC*), the differences were not statistically significant (*p* > 0.05).

The detection rates of biofilm‐related genes across strains with varying biofilm formation capabilities are shown in Table 4. No significant correlation was observed between biofilm formation ability and the presence of biofilm‐related genes (*p* > 0.05).

**TABLE 4:** Biofilm‐related gene presence in strains with varying biofilm formation abilities.

Biofilm gene	Biofilm formation status			*p*
	Strong (*N*, %)	Moderate (*N*, %)	Weak (*N*, %)	
*icaAD*	7 (100)	19 (100)	14 (93.33)	0.537^a^
*icaBC*	7 (100)	18 (94.74)	15 (100)	1.000^a^
*sasG*	7 (100)	15 (78.95)	12 (80.00)	0.641^a^
*sasC*	7 (100)	17 (89.47)	13 (86.67)	1.000^a^
*fnbpA*	7 (100)	19 (100)	15 (100)	—
*pls*	2 (28.57)	2 (10.53)	1 (6.67)	0.299^a^

*Note:* ^a^Indicates the presence of biofilm‐related genes in isolates exhibiting varying biofilm‐forming capacities, as determined by Fisher's exact test.

A total of 90.24% (37/41) of isolates harbored at least one of the five enterotoxin genes. The *sea* gene exhibited the highest carriage rate at 87.80% (36/41); while the carriage rates for *seb* to *see* genes ranged from 12% to 32% (Table 5).

**TABLE 5:** Distribution of enterotoxin genes in MRSA and MSSA strains.

Genes	MRSA (*n* = 8)	Percent (%)	MSSA (*n* = 33)	Percent (%)	Total (*n* = 41)	Percent (%)	*p*
*sea*	6	75.00	30	90.91	36	87.80	0.246^b^
*seb*	2	25.00	10	30.30	12	29.27	1.000^a^
*sec*	1	12.50	12	36.36	13	31.71	0.380^a^
*sed*	1	12.50	5	15.15	6	14.63	1.000^a^
*see*	1	12.50	4	12.12	5	12.20	1.000^b^

*Note:* ^a,b^Indicates distribution of enterotoxin genes among MRSA and MSSA strains, as determined by continuity correction (two‐sided) and Fisher's exact test.

Except for the *see* gene, MSSA strains showed a higher prevalence of *sea* to *sed* enterotoxin genes than MRSA strains; however, these differences were not statistically significant (*p* > 0.05). The PVL gene was detected in 25.00% (2/8) of MRSA strains and 9.09% (3/33) of MSSA strains, with no significant difference in the PVL gene carriage rate between the two groups (*p* > 0.05). *BlaZ* prevalence differed marginally between MRSA (6/8, 75.00%) and MSSA (22/33, 66.67%) isolates (*p* > 0.05), while overall carriage reached 68.29% (28/41). Concordance between the *blaZ* genotype and penicillin resistance phenotype was observed in 87.50% (28/32) of strains.

### 3.5 Enterotoxin Production in *S. aureus* Isolates

Fifteen isolates (36.59%) tested positive for at least one enterotoxin. Among the five enterotoxins screened, sea demonstrated the highest detection rate (34.15%, 14/41), followed by sec (17.07%, 7/41) and seb (12.20%, 5/41), whereas sed and see were not detected. Seven distinct enterotoxin profiles were identified: sea‐sec (9.76%, 4/41), sea‐seb (7.32%, 3/41), sea (7.32%, 3/41), sea‐see, sec‐see, sea‐seb‐sec, and sea‐sec‐see (each 2.44%, 1/41).

### 3.6 Molecular Characteristics of Isolated Bacterial Strains

**FIGURE 4:**
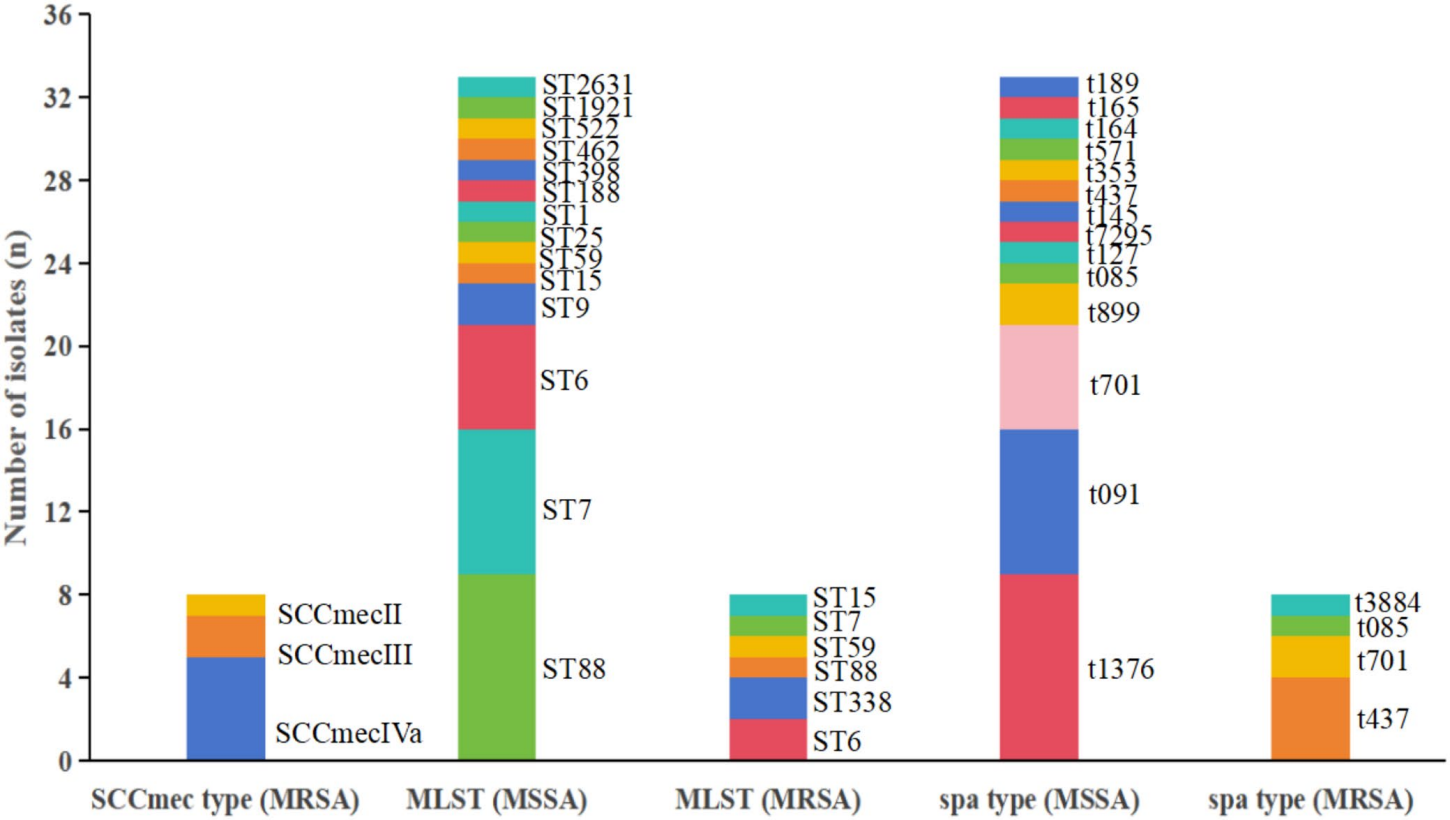
Molecular characterization of food‐derived *Staphylococcus aureus* isolates (*n* = 41) from northwest Hubei, China. MLST, multilocus sequence typing; SCCmec, Staphylococcal Cassette Chromosome mec; *spa*, staphylococcal protein A; ST, sequence type.

**FIGURE 5:**
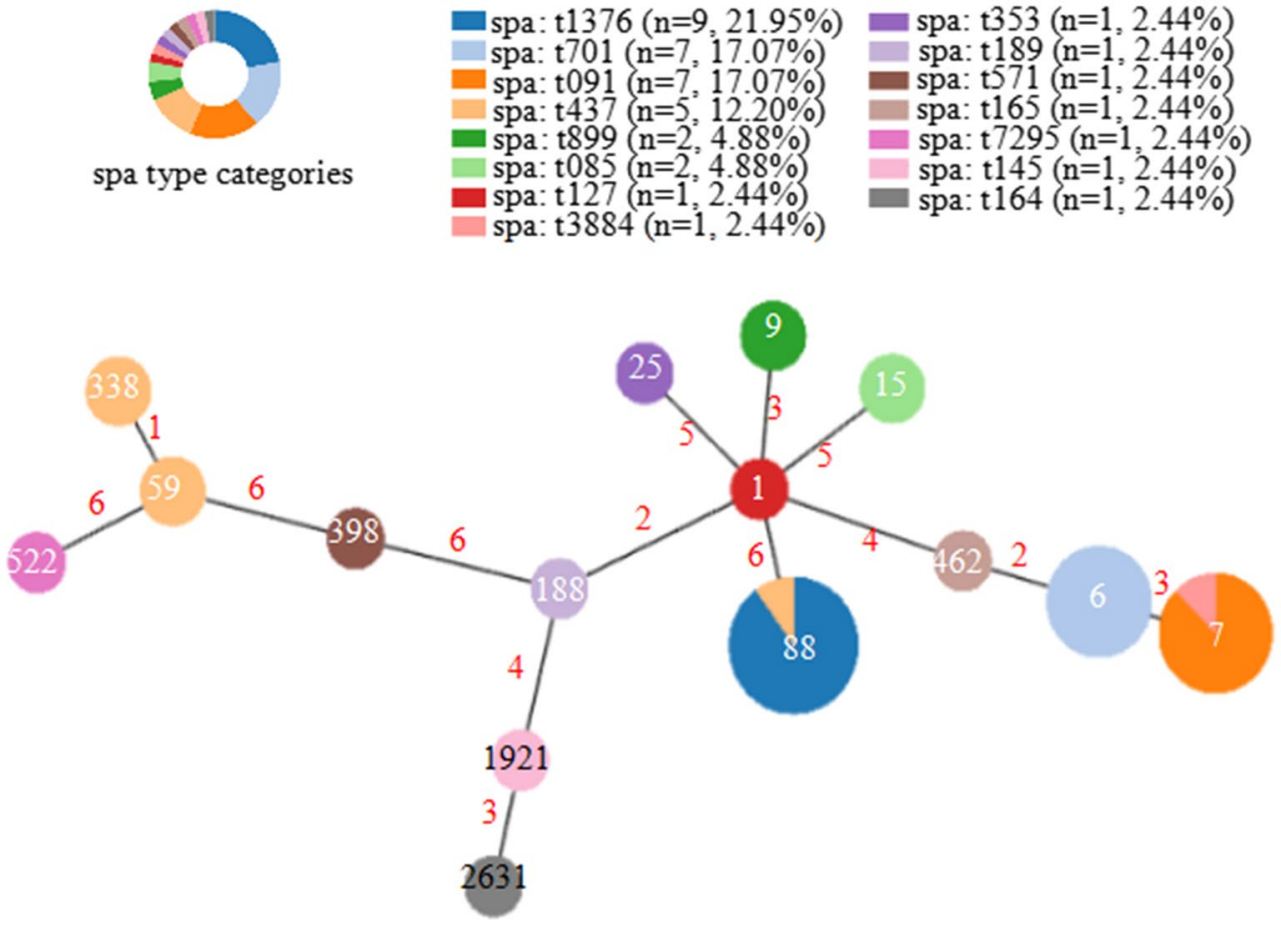
Minimum‐spanning tree constructed with PHYLOViZ online depicting genetic relationships among 41 foodborne *Staphylococcus aureus* strains isolated from northwest Hubei Province, China. Each node represents a unique ST type. The size of each node corresponds to the number of strains. The length between two nodes reflects the genetic distance between the two bounding ST types. The color partition of each disc corresponds to the proportion of the SPA types. Figures on the nodes are ST numbers.

The molecular characteristics of 41 isolates are summarized in Figure 4. Among the MRSA strains, SCCmec typing revealed that 62.50% (5/8) were SCCmec IVa, 25% (2/8) were SCCmec III, and 12.50% (1/8) were SCCmec II. A total of 15 sequence types (STs) were identified across all isolates. The eight MRSA strains included six STs: ST6 and ST338 were the most common (each 25%, 2/8), followed by ST59, ST88, ST7, and ST15, each represented by a single strain (12.50%, 1/8). In contrast, the 33 MSSA strains comprised 14 STs, with ST88, ST7, and ST6 predominating, accounting for 27.27% (9/33), 21.21% (7/33), and 15.15% (5/33) of isolates, respectively. Other STs included ST9 (6.06%, 2/33), and a single strain each of ST15, ST59, ST25, ST1, ST188, ST398, ST462, ST522, ST1921, and ST2631 (3.03%, 1/33). Based on the International MLST database (https://pubmlst.org/) and goeBURST analysis, all STs were assigned to eight clonal complexes (CCs) and three unique haplotypes (Figure 5): CC88‐ST88 (24.39%, 10/41), CC7‐ST7 (19.51%, 8/41), CC5‐ST6/ST462 (19.51%, 8/41), CC59‐ST59/ST338 (9.76%, 4/41), CC15‐ST15 (4.88%, 2/41), CC1‐ST9/ST1/ST188 (9.76%, 4/41), CC398‐ST398 (2.44%, 1/41), CC20‐ST1921 (2.44%, 1/41), ST25 (2.44%, 1/41), ST522 (2.44%, 1/41), and ST2631 (2.44%, 1/41). Minimum spanning tree analysis (Figure 5) indicated that foodborne isolates in this study display a relatively diverse population structure.

A total of 15 *spa* types were identified (Figures 4 and 5). Among the MRSA strains, four *spa* types were detected, with t437 being the most prevalent (50%, 4/8), followed by t701 (25%, 2/8), t7085 (12.50%, 1/8), and t3884 (12.50%, 1/8). In MSSA strains, 14 *spa* types were identified, with t1376, t091, and t701 predominating, accounting for 27.27% (9/33), 21.21% (7/33), and 15.15% (5/33), respectively. Other types included t899 (6.06%, 2/33), and one strain each of t437, t085, t127, t7295, t145, t353, t571, t164, t165, and t189 (3.03%, 1/33).

The relationships between molecular characteristics, biofilm formation, and antimicrobial resistance are presented in Table 6. The dominant epidemic clones were CC88‐ST88‐t1376/t437, CC7‐ST7‐t091/t3884, and CC5‐ST6/ST462‐t701/t165, which accounted for 63.41% (26/41) of the isolates. All t1376 strains were ST88 (100%, 9/9), while only 1 out of 32 other *spa* type strains was ST88 (3.13%). All t091 strains were ST7 (100%, 7/7), with only 1 ST7 strain among the 34 other *spa* type strains (2.94%). All t701 strains were ST6 (100%, 7/7), with no ST6 strains present in any other *spa* type. A significant correlation was observed between these MLST and *spa* type groupings (*p* < 0.01, chi‐squared test with continuity correction).

**TABLE 6:** Relationship of molecular characteristics, biofilm formation, specimen source, and antimicrobial resistance of *Staphylococcus aureus* strains.

CC	ST	*spa* type	Biofilm formation status (*n*)	MSSA/MRSA (SCC *mec*)	Isolates (*n*)	Resistance profile (*n*)
CC88	ST88	t437	Moderate (1)	MRSA (IVa)	1	PEN(1)FOX(1)ERY(1)CLI(1)CHL(1)SM(1)
	ST88	t1376	Strong (1)/moderate (5)/weak (3)	MSSA	9	PEN(8)ERY(1)TCY(4)DOX(1)CLI(1)
CC7	ST7	t3884	Weak (1)	MRSA (III)	1	PEN(1)FOX(1)ERY(1)TCY(1)GEN(1)CIP(1)SXT(1)
	ST7	t091	Moderate (2)/weak (5)	MSSA	7	PEN(4)ERY(2)TCY(4)DOX(1)CLI(1)
CC5	ST6	t701	Moderate (2)	MRSA (IVa)	2	PEN(2)FOX(2)ERY(1)TCY(2)DOX(2)CLI(1)CHL(2)
	ST6	t701	Strong (1)/moderate (2)/weak (2)	MSSA	5	PEN(4)CLI(2)
	ST462	t165	Weak (1)	MSSA	1	None
CC59	ST338	t437	Strong (2)	MRSA (III/IVa)	2	PEN(2)FOX(2)ERY(2)TCY(2)CLI(1)CHL(1)SM(2)
	ST59	t437	Strong (1)	MRSA (IVa)	1	PEN(1)FOX(1)ERY(1)DOX(1)SM(1)
	ST59	t437	Moderate (1)	MSSA	1	None
CC15	ST15	t085	Strong (1)	MRSA (II)	1	PEN(1)FOX(1)ERY(1)TCY(1)DOX(1)CLI(1)CIP(1)SXT(1)RIF(1)
CC1	ST9	t899	Moderate (1)/weak (1)	MSSA	2	PEN(1)TCY(2)
	ST1	t127	Strong (1)	MSSA	1	None
	ST188	t189	Moderate (1)	MSSA	1	PEN(1)
CC15	ST15	t085	Moderate (1)	MSSA	1	PEN(1)ERY(1)DOX(1)
CC398	ST398	t571	Weak (1)	MSSA	1	PEN(1)ERY(1)TCY(1)DOX(1)CHL(1)SM(1)
CC20	ST1921	t145	Moderate (1)	MSSA	1	PEN(1)CHL(1)
—	ST25	t353	Moderate (1)	MSSA	1	PEN(1)ERY(1)TCY(1)DOX(1)CHL(1)
—	ST522	t7295	Weak (1)	MSSA	1	PEN(1)
—	ST2631	t164	Moderate (1)	MSSA	1	PEN(1)

Among the CC59‐ST59/338 isolates, 75% (3/4) exhibited strong biofilm formation capabilities, compared to only 10.81% (4/37) of isolates from other lineages, indicating a significantly higher propensity for strong biofilm formation in CC59‐ST59/338 strains (*p* < 0.01). Conversely, 75% (6/8) of CC7‐ST7 strains formed weak biofilms, compared to 27.27% (9/33) of strains from other lineages, suggesting a strong association between CC7‐ST7 and weak biofilm formation (*p* < 0.01).

Among MRSA strains, the CC15‐ST15‐t085 clone exhibited the highest antimicrobial resistance, displaying resistance to nine antibiotics. Other MRSA strains were resistant to 5–7 antibiotics. Among the MSSA strains, CC398‐ST398‐t571 and ST25‐t353 showed resistance to 6 and 5 antibiotics, respectively. The locally prevalent strains MSSA‐CC88‐ST88‐t1376 and MSSA‐CC7‐ST7‐t091 exhibited resistance to 2–5 antibiotics (penicillin, erythromycin, tetracycline, doxycycline, and clindamycin), while MSSA‐CC5‐ST6‐t701 was resistant to two antibiotics (penicillin and clindamycin). Other isolates, such as ST188‐t189, ST522‐t7295, and ST2631‐t164, were resistant only to penicillin, while ST462‐t165, ST59‐t437, and ST1‐t127 showed no resistance to any tested antibiotics.

## 4 Discussion

With the extensive use of antibiotics in clinical treatments and animal farming, *S. aureus* continues to evolve under antibiotic selection pressure, remaining prevalent across hospital settings, communities, and animal environments. Beyond clinical infections, *S. aureus* contaminates various food products, particularly meats, leading to foodborne infections and food poisoning (Li et al. 2020; Fessler et al. 2011; Crago et al. 2012) and posing a serious threat to public health.

In this study, we isolated 41 *S. aureus* strains from food samples collected in the northwest region of Hubei Province, yielding a detection rate of 13.53% (41/303). The highest detection rates were observed in fresh meat, frozen meat, and vegetarian salad samples, with prevalence rates of 24.62%, 22.22%, and 14.75%, respectively, consistent with reports showing high *S. aureus* prevalence in meat products and salads (Wu et al. 2018a; Liao et al. 2018).

Previous studies have reported significant variation in the prevalence of foodborne MRSA, with rates ranging from 0.5% to 29.5% (Liao et al. 2018; Wu et al. 2019; Guo et al. 2023). In our study, MRSA detection was 19.51% (8/41), lower than the clinical MRSA isolation rates of 40.5% locally and 29.4% nationwide in China during the same period (Tang et al. 2024; Zhang et al. 2023). This suggests that foodborne strains experience relatively lower environmental antibiotic pressure, contributing to a lower rate of MRSA formation.

This study revealed the highest MRSA detection rates in rice and noodle products (50%) and frozen meat (33.33%), while rates were lower in vegetarian salads (22.22%) and fresh meat (12.50%). No MRSA was detected in cooked meat (0/4). This contrasts with reports indicating higher MRSA isolation from cooked and fresh meats, which may reflect geographical differences, sample types, and substrate quantities used (Weese et al. 2010).

The isolates in this study demonstrated high resistance to penicillin, tetracycline, and erythromycin, consistent with the findings of Zhang et al. (2024). Notably, the resistance of foodborne isolates in this region to most antibiotics was lower than that of clinically isolated *S. aureus* strains, although tetracycline resistance was remarkably high at 43.9%, compared to 20.7% in clinical isolates during the same period (Tang et al. 2024). This elevated resistance to tetracycline may stem from its frequent use as a first‐line antibiotic in veterinary practice and widespread application in agriculture. The high proportion of meat samples in our study (46.20%, 140/303) supports this association, highlighting the role of antibiotic overuse in promoting resistance. Furthermore, MRSA strains exhibited significantly higher resistance to erythromycin, chloramphenicol, streptomycin, ciprofloxacin, and trimethoprim‐sulfamethoxazole than MSSA strains, with all MRSA strains classified as multidrug‐resistant. These findings align with previous reports by Li et al. (2022), suggesting that the prevalence of multidrug‐resistant strains results from indiscriminate antibiotic use and misuse (Syed et al. 2018). Controlling antibiotic application in agriculture and veterinary medicine to prevent the emergence and spread of multidrug‐resistant foodborne MRSA strains is an urgent need in this region.

*S. aureus* food poisoning is primarily associated with food contamination and enterotoxin secretion during food processing and preparation. As members of the superantigen family, staphylococcal enterotoxins can disrupt adaptive immunity by stimulating T cells, leading to the release of significant amounts of pro‐inflammatory cytokines (Bae et al. 2021; Grispoldi et al. 2019). To date, 27 types of staphylococcal enterotoxins (SEs) have been identified, including five classic types (*sea‐see*) and 22 novel types (*seg‐sel27*). The majority of staphylococcal food poisoning (SFP) outbreaks are caused by the five classic SEs (Zhang et al. 2024).

The prevalence of enterotoxin genes among foodborne *S. aureus* isolates can vary significantly based on the region and type of food source (Liao et al. 2018; Wu et al. 2019; Titouche et al. 2020). Wu et al. (2019) found that foodborne *S. aureus* strains commonly carry multiple enterotoxin genes, with *sec* showing the highest detection rate at 75%. Detection rates for *sea*, *seb*, *sed*, and *see* were reported at 63.89%, 50.93%, 12.96%, and 12.96%, respectively. In contrast, Liao et al. (2018) reported *sed* as the most prevalent enterotoxin gene (57.77%), with detection rates for other enterotoxin genes ranging from 12% to 50%. In the present study, 90.24% (37/41) of isolates carried at least one enterotoxin gene, with *sea* being the most prevalent at 87.8%, which is consistent with the 81.08% detection rate reported by Chen and Xie (2019). MRSA strains exhibited a lower carriage rate of enterotoxins compared to MSSA strains, aligning with findings from Wang et al. (2014). Significant regional and food‐type variations in *S. aureus* enterotoxin detection rates have been reported (Wang et al. 2018; Tarekgne et al. 2016). In this study, the enterotoxin protein detection rate (36.59%, 15/41) aligned with findings by Lv et al. (2021). The lower expression levels compared to gene carriage rates may stem from environmental influences, genetic regulation, protein stability limitations, or insufficient sensitivity of detection methods.

These results indicate that foodborne *S. aureus* strains in this region harbor a high prevalence of enterotoxin genes, particularly among MSSA strains. This genetic profile raises concerns about the potential risk of food poisoning (due to enterotoxin production) if contaminated foods are stored under permissive conditions.

The PVL gene, a virulence factor linked to severe infections, was detected in 12.20% (5/41) of isolates in this study, a rate consistent with the 11.6% prevalence reported by Yang et al. (2018) and lower than the 16.73% reported by Liao et al. (2018) for foodborne isolates in southwestern China. Among MRSA strains, the PVL gene detection rate was 25% (2/8), which aligns with the 24.07% rate observed by Wu et al. (2019) for foodborne MRSA and is higher than the 18.6% detection rate among locally sourced clinical *S. aureus* strains (Tang et al. 2018).

All strains in this study exhibited biofilm‐forming ability, with the majority (46.34%) producing moderate‐strength biofilms. MRSA strains demonstrated a significantly higher capacity for strong biofilm formation than MSSA strains, aligning with the findings of Ballah et al. (2022). Previous research has shown that biofilm formation can vary by food source, with isolates from different food types displaying distinct biofilm‐forming capacities (Ballah et al. 2022; Chen, Xie, et al. 2020). In this study, strains from frozen meat samples were more likely to form strong biofilms, whereas strains from cooked meat were predominantly weak biofilm producers.

The relationship between biofilm formation capability and biofilm‐associated genes in *S. aureus* depends on multiple factors, including the strain's resistance profile, genetic background, and isolation source. Yu et al. (2024) demonstrated that *icaA*, *icaB*, *icaC*, *icaD*, *icaR*, *sdrE*, and *ebp* are associated with strong biofilm formation in clinically derived MRSA strains. Chen, Tang, et al. (2020) reported that *S. aureus* strains with strong biofilm‐forming capacity exhibit a higher prevalence of the *clfA*, *clfB*, *fnbA*, and *sdrC* genes, with ST59 and ST188 strains showing particularly strong biofilm‐forming ability. In contrast, studies on strains isolated from clinical, animal, and food poisoning‐related samples (Tang et al. 2024, 2013) found no significant correlation between biofilm‐forming capability and the presence of biofilm‐associated genes. In the current study, MRSA strains exhibited stronger biofilm formation than MSSA strains; however, no direct correlation was observed between biofilm formation and biofilm‐associated genes. This suggests that biofilm production in *S. aureus* is governed by a complex regulatory network. Therefore, biofilm formation cannot be reliably predicted solely by the presence of specific biofilm genes. Future studies should account for differences in strain genetic background and isolation source, combined with transcriptomic profiling (e.g., RT‐qPCR) and investigations into multi‐gene cooperative networks, to elucidate the molecular mechanisms underlying phenotypic variation in *S. aureus* biofilms.

Regional differences significantly impact the molecular epidemiology of foodborne *S. aureus* . For instance, ST72 and ST1 are predominant in South Korea (Shin et al. 2016), while ST81 and ST45 are more common in Japan (Sato'o et al. 2014). In China, the main types include ST188, ST6, ST7, ST5, and ST59 (Chen and Xie 2019; Zhou et al. 2022, 2024). In Hangzhou, ST6‐t304 is the most frequent foodborne *S. aureus* clone, comprising 40.54% of all isolates (Chen and Xie 2019). In southwestern China, ST6‐t701, ST7‐t091, ST59‐t437, and ST5‐t002 are the prevalent foodborne clones, accounting for 13.15%, 12.75%, 9.96%, and 7.57% of strains, respectively. Additionally, among foodborne MRSA isolates, ST6‐SCCmecIV‐t701 (36.5%) is the most common clone, followed by ST59‐SCCmecV‐t437 (20.3%), ST5‐SCCmecIV‐t002 (12.2%), and ST59‐SCCmecIV‐t437 (12.2%) (Liao et al. 2018). Research by Gu et al. (2023) indicates that post‐COVID‐19, ST7 has become a predominant *S. aureus* clone in Wuhan, with increased SCCmec and virulence gene carriage rates. This shift may be associated with intensified disinfectant use during the pandemic, creating selective pressure that promotes *S. aureus* adaptation and evolution in response to antibiotics, immune defenses, and environmental shifts.

The ST6 clone, which is prevalent in food, nasopharyngeal samples from healthy individuals, and high‐traffic public environments such as airports and train stations in Guangdong Province, is likely to spread within community and public settings (Wu et al. 2018b; Chen et al. 2018).

Among the eight MRSA strains isolated in this study, SCCmecIVa was the predominant type, accounting for 62.50% (5/8), consistent with previous reports (Liao et al. 2018; Guo et al. 2023; Lawal et al. 2022), and suggesting that SCCmecIV is the most common foodborne MRSA type in this region. The ST88‐t437 clone was identified as the primary epidemic clone (24.39%, 10/41). ST88 is widely prevalent across the African continent (Lawal et al. 2022) and has been reported as a dominant community‐associated clone in China (Wang et al. 2016). However, ST88 has not been widely documented as a major foodborne clone in other regions of China, highlighting the need to monitor the transmission dynamics of this strain between foodborne and community‐associated infections in this region.

Overall, the isolates in this study exhibited a relatively diverse population structure. However, certain predominant clones demonstrated elevated isolation frequencies in specific food categories. ST88 strains were recovered from 31.25% (5/16) of fresh meat products, exceeding their prevalence in frozen meats (16.67%, 1/6) and bean products (0/4). Similarly, ST7 strains showed significantly higher isolation rates in fresh meats (18.75%, 3/16) compared to frozen meat products, where they were not detected (0/6). Although these inter‐group differences lacked statistical significance, potential cross‐contamination events or localized clonal expansion within specific food matrices cannot be excluded. This study has several methodological limitations: the relatively small cohort size (*n* = 41 isolates), potential sampling bias due to uneven representation of food types (notably, meat products constituted 50.83% [154/303] of total samples), and the absence of functional validation experiments for antibiotic resistance and biofilm formation mechanisms. Future investigations should incorporate wider geographic sampling, more balanced food category representation, larger cohort sizes, and functional validation studies to strengthen the analytical robustness of molecular epidemiological characteristics in regional foodborne *S. aureus* strains.

In this study, CC59‐ST59/338 strains exhibited a notable capacity for strong biofilm formation, and both PVL‐positive MRSA strains belonged to CC59‐ST338. CC59 clones are widely distributed and commonly associated with community‐acquired MRSA (CA‐MRSA) (Tang et al. 2018; Li et al. 2013). PVL‐positive *S. aureus* strains can cause severe infections such as necrotizing pneumonia, bloodstream infections, and soft tissue infections, which present a risk of transmission to humans through the food chain (Hammad et al. 2012). Thus, it is crucial to implement effective strategies to prevent foodborne infections and limit the transmission of PVL‐positive ST338/t437 strains.

This study also highlighted associations between molecular types, biofilm formation capacity, and antibiotic resistance. For example, 90% (9/10) of ST88 strains were identified as t1376, 87.50% (7/8) of ST7 strains as t091, and 100% (7/7) of ST6 strains as t701, which is consistent with clinical strain profiles in this region (Tang et al. 2024). The CC59‐ST59/338 strains showed a pronounced capacity for strong biofilm formation, whereas CC7‐ST7 strains were more likely to form weak biofilms. Among MRSA isolates, CC15‐ST15‐t085 demonstrated the highest level of antibiotic resistance, with resistance to nine antibiotics. Among MSSA strains, CC398‐ST398‐t571 and ST25‐t353 were the most resistant, with resistance to 5–6 antibiotics. Locally prevalent MSSA strains such as CC88‐ST88‐t1376, CC7‐ST7‐t091, and CC5‐ST6‐t701 exhibited lower resistance, with resistance to only three or fewer antibiotics, which is lower than the resistance reported by Zhou et al. (2022) for foodborne ST7‐t091 and ST6‐t701 strains to 6–7 antibiotics.

## 5 Conclusion

This study reveals a high prevalence of foodborne *S. aureus* in northwest Hubei Province, with MRSA strains displaying significantly elevated antibiotic resistance and biofilm‐forming capacities compared to MSSA strains. The high carriage rates of enterotoxin genes across isolates underscore the potential of these strains to contribute to foodborne illness. Among the predominant clones identified, CC88‐ST88‐t1376/t437, CC7‐ST7‐t091/t3884, and CC5‐ST6/ST462‐t701/t165 are particularly relevant as primary targets for monitoring and controlling foodborne *S. aureus* infections in the region. Notably, the ST88 clone appears to bridge transmission routes between foodborne sources and community settings, highlighting its importance in both epidemiological surveillance and infection prevention strategies. These findings enhance our understanding of the drug resistance profiles and molecular epidemiology of *S. aureus* in food sources, providing a foundation for more effective control measures. By identifying the specific strain types and their associated resistance traits, this study offers critical insights to guide regional food safety policies and public health interventions aimed at curbing the spread of multidrug‐resistant *S. aureus* through the food supply chain.

## Author Contributions

**Yitong Tang:** conceptualization (lead), data curation (lead), methodology (lead), writing – original draft (lead). **Yanyan Li:** conceptualization (supporting), methodology (supporting), writing – review and editing (supporting). **Na Xiao:** data curation (supporting), investigation (supporting), methodology (supporting), writing – review and editing (supporting). **Lu Wang:** investigation (supporting), methodology (supporting). **Shichao Li:** investigation (supporting), validation (supporting). **JiuMing Zou:** validation (supporting). **Yanmin Yu:** validation (supporting). **Jinhui Shao:** investigation (supporting). **Ming Jiang:** investigation (supporting).

## Ethics Statement

The authors have nothing to report.

## Conflicts of Interest

The authors declare no conflicts of interest.

## Data Availability

The data that support the findings of this study are available from the corresponding author upon reasonable request.
